# Amelioration of tic disorder by Jujuboside A via gut microbiota remodeling and intestinal 5-HT signaling

**DOI:** 10.3389/fnins.2026.1760647

**Published:** 2026-05-18

**Authors:** Fei Fan, Bo Wang, Ru Jia, Jing Lyu, Fei Han

**Affiliations:** Department of Pediatrics, Guang’anmen Hospital, China Academy of Chinese Medical Sciences, Beijing, China

**Keywords:** 5-HT, gut microbiota, Jujuboside A, microbiota-gut-brain axis, tic disorder

## Abstract

**Background:**

Tic disorder (TD) is a common chronic neuropsychiatric condition manifesting during childhood and adolescence. Jujuboside A (JuA) may alleviate TD symptoms; however, the mechanisms underlying its therapeutic effects remain unclear.

**Methods:**

We established a rat model of TD and used histological techniques to evaluate the effects of JuA on pathological changes. We also measured 5-hydroxytryptamine (5-HT) and 5-hydroxyindoleacetic acid (5-HIAA) levels and assessed tryptophan hydroxylase 1 (TPH1) mRNA expression. Finally, we analyzed the gut microbiota composition in fecal samples using 16S rRNA metagenomic sequencing.

**Results:**

JuA administration alleviated pathological changes in rats with TD, increased 5-HT and 5-HIAA levels, and upregulated TPH1 mRNA expression. Compared with no treatment, JuA treatment increased the proportion of *Bacteroidia*, *Muribaculaceae*, *Bacteroidales*, and *Bacteroidota*, while reducing that of *Bacilli*, *Lactobacillaceae*, *Lactobacillus*, *Lactobacillales*, and *Firmicutes*.

**Conclusion:**

These findings indicate that JuA mitigates TD progression, potentially by remodeling the gut microbiota and regulating 5-HT levels.

## Background

Tic disorders (TD) are neuropsychiatric syndromes characterized by involuntary, repetitive, sudden, and non-rhythmic movements of one or more parts of the body (Müller-Vahl et al., 2022). Common clinical features include head shaking, shoulder shrugging, eyebrow raising, blinking, facial muscle twitching, abnormal throat sounds, and coprolalia (the use of obscene language) (Stiede and Woods, 2020). TD onset is typically observed at age 5–6 years; it peaks in severity between ages 10 and 12 and gradually subsides in early adulthood (Pringsheim et al., 2019b). Most patients experience symptom remission during adulthood. Furthermore, more than 50% of patients with TD present with varying degrees of mental or behavioral comorbidities (Pringsheim et al., 2019a). The most common mental comorbidities are attention deficit hyperactivity disorder, obsessive-compulsive disorder, anxiety, and depression, all of which impair psychological and social functioning (Yang et al., 2020).

The pathogenesis of TD is complex and remains poorly understood. Symptom heterogeneity and the high prevalence of comorbidities limit the efficacy of clinical treatments (Yael et al., 2015). Current treatments for TD primarily consist of psychological interventions, cognitive behavioral therapy, and pharmacotherapy (Tagwerker Gloor and Walitza, 2016). Additionally, noninvasive neuroregulation and deep brain stimulation have been used for TD management (Martino and Pringsheim, 2018). However, clinical studies have shown that the efficacy of psychological and behavioral interventions is often limited. The long-term efficacy of existing treatments is suboptimal, and relapse frequently occurs following drug discontinuation. Furthermore, these medications may cause adverse reactions, including drowsiness, concentration deficits, memory impairment, and extrapyramidal symptoms (Knight et al., 2012).

Jujuboside A (JuA), an active compound derived from *Semen Ziziphi Spinosae*, exhibits neuroprotective properties through its antioxidative and anti-inflammatory effects (Yang et al., 2025; Fan et al., 2022). Recent studies have shown that JuA may attenuate stereotypical behaviors in rats with TD by modulating glutamate and gamma-aminobutyric acid levels in the extracellular fluid of the caudate putamen. This effect may be mediated by the upregulation of Glutamate Transporter-1 and Glutamate/Aspartate Transporter protein expression (Fan et al., 2025). However, the precise underlying mechanism remains unclarified.

The microbiota-gut-brain axis (MGBA) is a bidirectional communication pathway between the gut and brain (Wang et al., 2023). It has been implicated in the pathophysiology of neuropsychiatric disorders, including Alzheimer’s disease, Parkinson’s disease, autism, and attention deficit hyperactivity disorder (He et al., 2023; Cui et al., 2024; Olsen and Hicks, 2020; Cai et al., 2025). Moreover, the MGBA has been implicated in the pathogenesis of TD (Xi et al., 2021; Tagi et al., 2024). 5-Hydroxytryptamine (5-HT) is a neurotransmitter found in the central nervous system (CNS) and the digestive tract (Sanger, 2008). The gut microbiota regulates its synthesis, metabolism, and physiological functions (Yano et al., 2015). Approximately 95% of total body 5-HT is synthesized by intestinal enterochromaffin cells and enteric neurons, whereas the remaining 5% is produced in the CNS (Park et al., 2015).

In this study, we investigated alterations in the gut microbiota and 5-HT levels in a rat model of TD treated with JuA and normal control rats. We utilized 16S rRNA whole-gene sequencing to provide data supporting potential clinical applications.

## Methods

### Animal model and treatment

Adult male Sprague Dawley rats (8 weeks old; 200–230 g) were obtained from Liaoning Changsheng Biotechnology Co., Ltd. and randomly assigned to the control (Group A, *n* = 6) and TD model (*n* = 24) groups. Baseline body weights did not differ significantly between the groups (*p* > 0.05). All experimental and animal care procedures were approved by the Chinese Academy of Traditional Chinese Medicine Guang’anmen Hospital (approval No. IACUC-GAMH-2025-019). The study was conducted in accordance with the National Institutes of Health *Guide for the Care and Use of Laboratory Animals* and reported following the Animal Research: Reporting of *In Vivo* Experiments guidelines. Rats were housed in a controlled environment (25 ± 1 °C; 45–55% relative humidity) under a 12-h light/dark cycle with *ad libitum* access to food and water. The TD model was established according to a previously described method (Diamond et al., 1982). Briefly, 3,3′-iminodipropionitrile (IDPN) was mixed with 0.9% NaCl, to create a solution with a concentration of 30 mg/mL. To induce the TD model, adult Sprague Dawley rats received intraperitoneal injections of IDPN (250 mg/kg) once daily for 7 consecutive days. Control rats received equivalent volumes of normal saline.

### JuA preparation and treatment

JuA was obtained from Beijing Solarbio Technology Co., Ltd. and dissolved in 0.9% NaCl to a concentration of 1 mg/mL. Tiapride was dissolved in 0.9% NaCl to a concentration of 3.125 mg/mL (Fan et al., 2025). After 7 days of model induction, the model group was subdivided into four subgroups: TD model (Group B, *n* = 6), low-dose JuA (Group C, administered at 5 mg/kg), high-dose JuA (Group D, administered at 10 mg/kg), and tiapride (Group E, administered at 31.25 mg/kg). Rats received JuA via oral gavage once daily for 3 weeks. Rats in Groups A and B received an equivalent volume of normal saline once daily for 3 weeks. During this period, rats had *ad libitum* access to food and water and were maintained in a comfortable living environment to ensure the smooth progression of the experiment and data reliability.

Following drug administration and behavioral testing, blood samples (approximately 1.5 mL) were collected from the tail vein into EDTA-coated tubes. No anesthesia was used during blood collection from the tail vein. Samples were centrifuged at 5000 × *g* for 10 min at 4 °C to separate plasma, which was aliquoted into Eppendorf tubes and stored at −80 °C. Throughout the experiment, the health of the rats was closely monitored, and no accidental deaths occurred. For euthanasia, rats were placed in a pre-filled CO₂ chamber. The CO₂ was introduced at a flow rate sufficient to displace 30–40% of the chamber volume per minute. Animals remained in the chamber for at least 2 min after respiratory arrest. Death was confirmed by the absence of spontaneous breathing, followed by cervical dislocation. Disposable sterile forceps were used to collect 2–3 fecal pellets from the colon of each rat. Samples were placed in sterile cryovials and snap-frozen in liquid nitrogen for sequencing. Colon tissue sections were fixed in 10% neutral-buffered formalin for 24 h for pathological evaluation; the remaining sections were used for real-time quantitative reverse-transcription polymerase chain reaction (qRT-PCR).

### Behavior analysis

Behavioral assessments were conducted every 7 days throughout the experiment, always between 9:00 a.m. and 12:00 p.m. to minimize circadian variability. All tests were performed in a dedicated quiet room with controlled lighting (~100 lux at cage level), temperature (25 ± 1 °C), and relative humidity (45–55%). White noise (60 dB) was continuously played to mask external sounds. Following a 5-min habituation period in an observation cage (40 cm × 40 cm × 40 cm), each rat was observed for 5 min under double-blind conditions (two independent observers blinded to group allocation, scoring from video recordings). Locomotor activity and stereotyped behaviors were scored based on previously established protocols with slight modifications (Al Kadasah et al., 2009; Zhu et al., 2022). The scoring system was defined as follows: 0 points: no abnormal behavior; 1 point (mild): occasional head shaking or sniffing lasting <30% of the observation period; 2 points (moderate): frequent intermittent circling or grooming occupying 30–60% of the period; 3 points (severe): continuous circling, jumping, or biting occupying >60% of the period; 4 points (extreme): incapacitating stereotypies with no response to stimuli. After each test, the observation cage was thoroughly cleaned with 70% ethanol to eliminate any olfactory or behavioral cues that might influence subsequent rats.

### qRT-PCR

Total RNA was isolated from colon tissue using TRIpure reagent (BioTeke). RNA concentration was quantified using a NanoDrop One UV spectrophotometer (Thermo). RNA was reverse-transcribed to cDNA using the All-in-One First-Strand SuperMix (Guangdong Meiji Biotechnology Co., Ltd.). PCR amplification was performed using 2 × Fast Taq Plus PCR Master Mix (Biosharp) on a Pangaea 3 fluorescence quantitative PCR instrument (Aperbio). Relative expression was calculated using the 2^−ΔΔCt^ method, with *β*-actin serving as the endogenous control. The primer sequences are listed in Table 1.

**Table 1:** Primer sequences.

Primer name	Sequences
*HT1AR* forward	5′-GCTCATTGGCTTTCTCATCT-3′
*HT1AR* reverse	5′-CGTTCAGGCTCTTCTTGG-3′
*HT2AR* forward	5′-TGATATGCTGCTGGGTT-3′
*HT2AR* reverse	5′-ATGATGGATGCCGTAGA-3′
*Tph1* forward	5′-TTCACTGTGGAGTTTGGACT-3′
*Tph1* reverse	5′-TTCACACACTGGGCCA-3′
β-actin forward	5′-TGTCACCAACTGGGACGATA-3′
β-actin reverse	5′-GGGGTGTTGAAGGTCTCAAA-3′

### Enzyme-linked immunosorbent assays (ELISA)

Colon tissue samples were weighed and homogenized in PBS (1:9 w/v). Samples were mechanically homogenized on ice and centrifuged at 2795 × *g* for 5 min. The supernatant was collected, and levels of 5-HT, 5-hydroxyindoleacetic acid (5-HIAA), and tryptophan hydroxylase 1 (TPH1) were quantified using specific ELISA kits (Wuhan Fine MEMS Co., Ltd.), following the manufacturer’s instructions. Optical density (OD) was measured at 450 nm using an ELX-800 microplate reader (BioTek), with correction at 570 nm. OD values were corrected by subtracting the OD570 from the OD450. The calibrated OD values were calibrated on the x-axis and the standard sample concentration on the y-axis; the corresponding concentrations were calculated based on the standard curve.

### Hematoxylin and eosin (HE) staining

Colon tissue samples were fixed in 4% paraformaldehyde, dehydrated with alcohol, cleared with xylene, embedded in paraffin, and sectioned at 5 μm. Sections were stained with hematoxylin for approximately 30 min, followed by eosin for 1 min. Slides were dehydrated, mounted with neutral balsam, and examined using an Olympus BX53 microscope coupled with a DP73 imaging system.

### Sequencing analysis of 16S rRNA of fecal gut microbiota

Fresh fecal samples were collected from each group and placed in sterile cryovials. Total microbial genomic DNA was extracted, and the V3-V4 hypervariable regions of the 16S rRNA gene were amplified using specific primers. The PCR products were purified and sequenced on the Illumina MiSeq platform. Raw sequencing reads were filtered to obtain high-quality clean reads. Paired-end reads were merged using FLASH, and chimeric sequences were removed. Merged sequences were clustered into OTUs using QIIME. Representative OTU sequences were taxonomically annotated, and composition was visualized. Alpha and beta diversity analyses were performed to explore differences in community structure among groups. Linear discriminant analysis (LDA) effect size (LEfSe) was performed to identify differentially abundant taxa among groups, with the LDA score threshold set at 3.0, which is a commonly used cutoff in microbiome studies to ensure biological relevance (Hao et al., 2024).

### Statistical analysis

Data were analyzed using GraphPad Prism 8.0 (GraphPad Software, Boston, MA, USA). Results are presented as mean ± standard deviation. Differences between multiple groups were assessed using one-way analysis of variance (ANOVA) followed by Tukey’s *post hoc* test. Behavioral scores (median with interquartile range) were analyzed using the Kruskal–Wallis test followed by Dunn’s post hoc test. Statistical significance was set at *p* < 0.05.

## Results

### Effects of JuA on the behavior of rats with TD

Activity and stereotypical behaviors were assessed on day 7 (post-induction) and day 21 (post-treatment). Post-induction activity and stereotypical behavior scores exceeded 2 points and were significantly higher than those of Group A, confirming successful model establishment. Following JuA treatment, scores in Groups C and D were significantly lower than those in untreated TD rats (Group B) (Table 2).

**Table 2:** Comparison of behavior scores among groups [median (Q1, Q3)] (unit: points).

Group	Post-induction	Post-treatment (Day 21)
Control (A)	0	0
Model (B)	3.50 (3.00, 4.00)	3.00 (3.00, 4.00)
Low-dose JuA (C)	3.50 (3.00, 4.00)	2.00 (2.00, 2.00)▲#
High-dose JuA (D)	3.50 (3.00, 4.00)	1.00 (1.00, 2.00)▲#
Tiapride (E)	3.00 (3.00, 4.00)	1.00 (1.00, 2.00)▲#

Behavioral scores were analyzed using Kruskal–Wallis test followed by Dunn’s post hoc test. ▲*p* < 0.05 vs. the same group after modeling; #*p* < 0.05 vs. the model group B at the same time point. JuA, Jujuboside A.

### Effects of JuA on the colon histology of rats with TD

**Figure 1:**
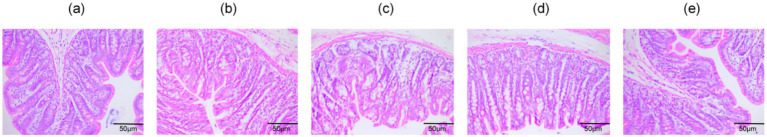
Alleviated pathological changes in rats with TD treated with JuA. Representative hematoxylin and eosin staining images of colon tissue (200 × magnification; scale bar = 50 μm): **(a)** Group A_2_; **(b)** Group B_2_; **(c)** Group C; **(d)** Group D; **(e)** Group E. Group A_2_: control-after treatment; Group B_2_: model-after treatment; Group C: low-dose JuA; Group D: high-dose JuA; Group E: tiapride; JuA, jujuboside A; TD, tic disorder.

Hematoxylin and eosin staining revealed that Group A exhibited intact colonic mucosal structure, characterized by neatly arranged goblet cells and normal glandular morphology. In contrast, Group B displayed significant goblet cell loss, glandular damage, and compromised mucosal integrity. Compared with that in Group B, pathological damage was attenuated in Groups C, D, and E, with Groups D and E exhibiting more obvious improvement. The therapeutic efficacy observed in Group D was comparable to that in Group E (Figure 1).

### JuA increases expression levels of 5-HT, 5-HIAA and TPH1 in rats with TD

**Figure 2:**
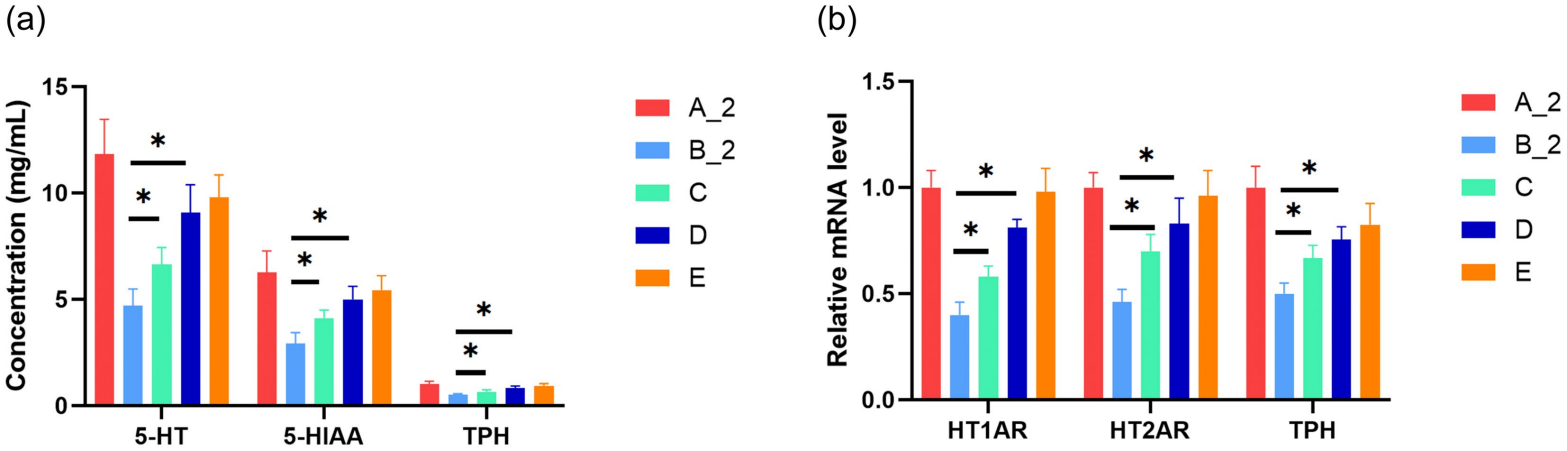
Increased expression levels of 5-HT, 5-HIAA, and TPH1 in rats with TD treated with JuA. **(a)** ELISA was used to determine changes of 5-HT, 5-HIAA, and TPH1 in each group. Data are expressed as mg/mL. **(b)** qPCR showing mRNA expression levels of *HT1AR*, *HT2AR*, and *Tph1* genes. Data are presented as the mean ± standard deviation (*n* = 6). *p < 0.05 compared with group B_2. Group A_2: control-after treatment; Group B_2: model-after treatment; Group C: low-dose JuA; Group D: high-dose JuA; Group E: tiapride. 5-HIAA, 5-hydroxyindoleacetic acid; 5-HT; 5-hydroxytryptamine; ELISA, enzyme-linked immunosorbent assays; *HT1AR*, 5-hydroxytryptamine 1A receptors gene; *HT2AR*, 5-hydroxytryptamine 2A receptors gene; JuA, Jujuboside A; TPH1, tryptophan hydroxylase 1.

We evaluated the effects of JuA on 5-HT, 5-HIAA, and TPH1 levels in the colon tissues of TD rats. We found that 5-HT, 5-HIAA, and TPH1 levels in colon tissues of Group B were significantly lower than those of Group A (Figure 2a). Similarly, mRNA expression of 5-hydroxytryptamine 1A receptors gene (*HT1AR*), 5-hydroxytryptamine 2A receptors gene (*HT2AR*), and *Tph1* was downregulated in Group B compared with Group A (Figure 2b). Compared with Group B, Groups C, D, and E exhibited significantly increased 5-HT, 5-HIAA, and TPH1 levels, as well as *HT1AR*, *HT2AR*, and *Tph1* mRNA expression. These increases were more pronounced in Groups D and E (Figure 2).

### Intestinal microbiota analysis of groups

**Figure 3:**
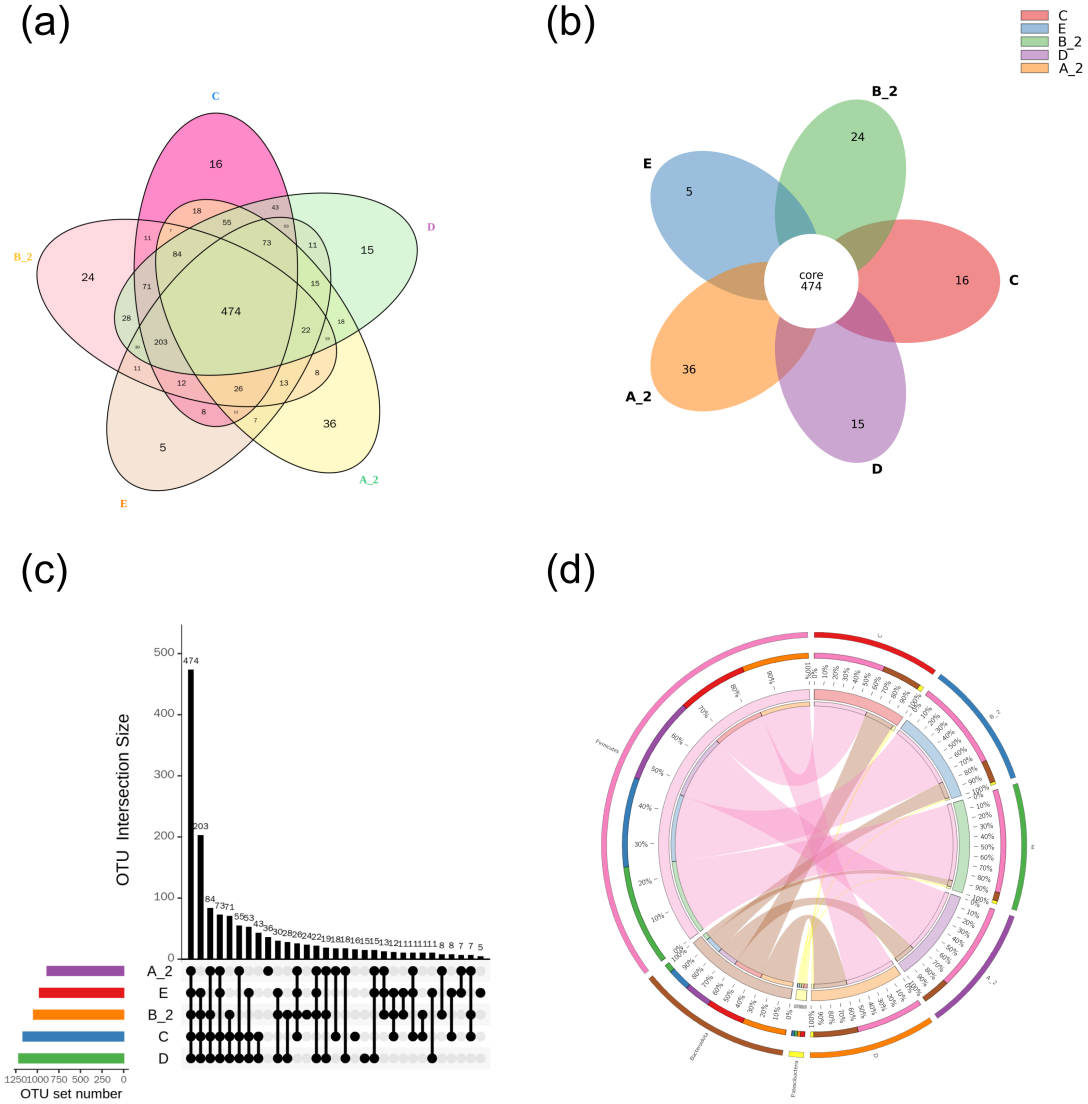
OTU clustering and analysis. OTUs were analyzed via the **(a)** Venn diagram, **(b)** flower diagram, **(c)** upset diagram, and **(d)** Circos plot of OTU distribution. Group A_2: control-after treatment; Group B_2: model-after treatment; Group C: low-dose JuA; Group D: high-dose JuA; Group E: tiapride. JuA, Jujuboside A; OTUs, operational taxonomic units.

We further investigated JuA-induced changes in gut microbiota. Fecal samples from Groups A, B, C, D, and E yielded 510, 498, 490, 489, and 479 OTUs, respectively. The respective number of unique OTUs was 36, 24, 16, 15, and 5, respectively (Figure 3).

**Figure 4:**
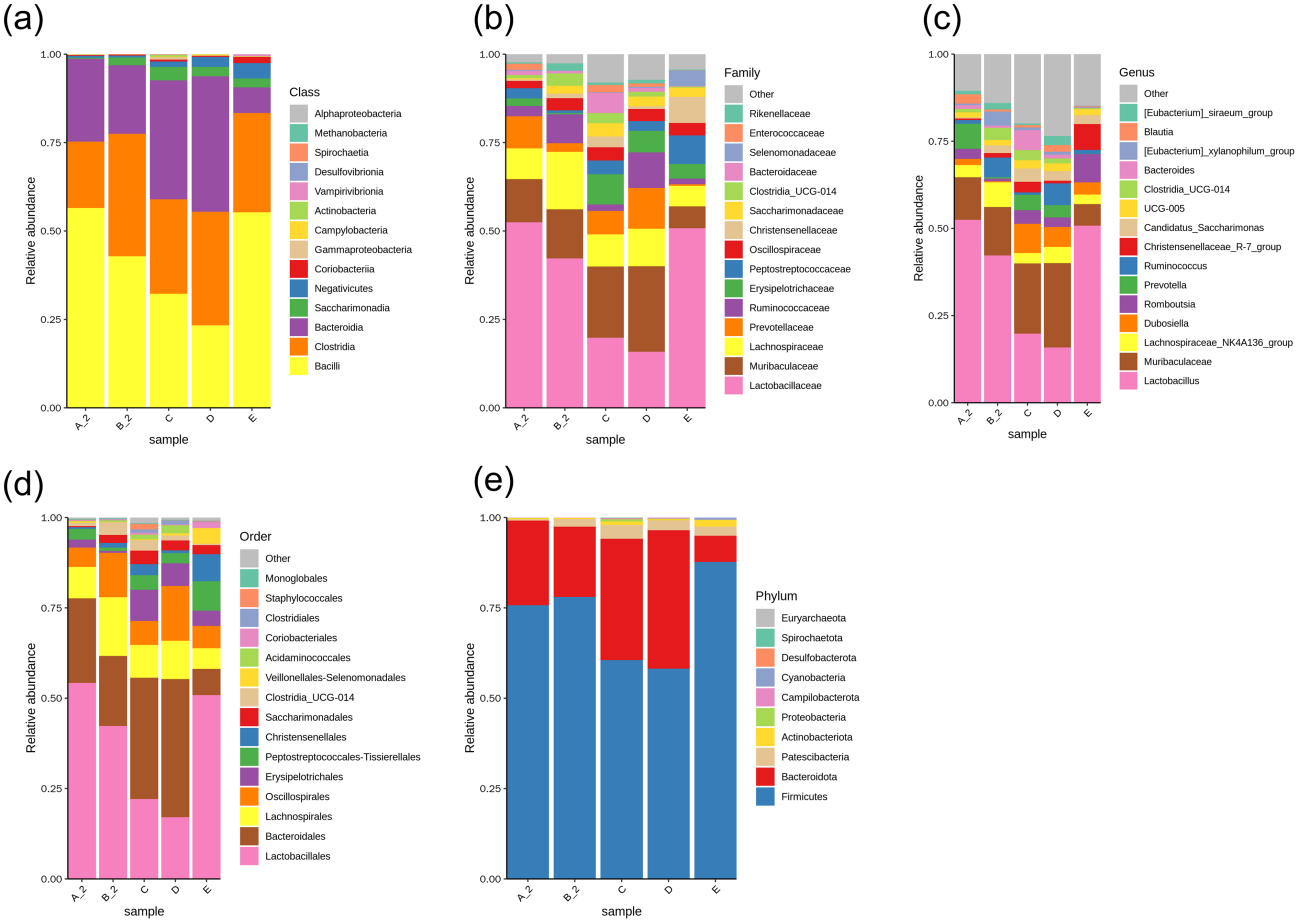
Species relative abundance bar chart. Bar charts showing composition at: **(a)** Class; **(b)** Family; **(c)** Genus; **(d)** Order; **(e)** Phylum. Group A_2: control- after treatment; Group B_2: model-after treatment; Group C: low-dose JuA; Group D: high-dose JuA; Group E: tiapride. JuA, Jujuboside A.

OTUs were clustered at a 97% similarity. Representative sequences were selected based on the highest abundance sequence within each cluster and taxonomically annotated. Species with the highest abundance at the Phylum, Class, Order, Family, and Genus levels were selected to generate relative abundance graphs (Figure 4). At the Class level, the relative abundance of *Bacilli* was lower in Group B than in Group A. Compared with Group B, *Bacilli* abundance decreased in Groups C and D but increased in Group E. Although *Bacteroidia* abundance did not differ significantly between Groups A and B, it increased in Groups C and D and decreased in Group E compared with in Group B (Figure 4a). At the Family level, *Lactobacillaceae* abundance was lower in Group B than in Group A. Compared with Group B, *Lactobacillaceae* decreased in Groups C and D but increased in Group E, whereas *Muribaculaceae* increased in Groups C and D and decreased in Group E (Figure 4b). At the Genus level, *Lactobacillus* was reduced in Group B compared with Group A. Relative to Group B, *Lactobacillus* decreased in Groups C and D but increased in Group E (Figure 4c). At the Order level, *Lactobacillales* decreased in Group B compared with Group A, with further reductions in Groups C and D but an increase in Group E. Conversely, *Bacteroidales* increased in Groups C and D but decreased in Group E (Figure 4d). At the Phylum level, *Firmicutes* followed the trend of *Lactobacillales*, while *Bacteroidota* increased in Groups C and Dand decreased in Group E compared with Group B (Figure 4e).

**Figure 5:**
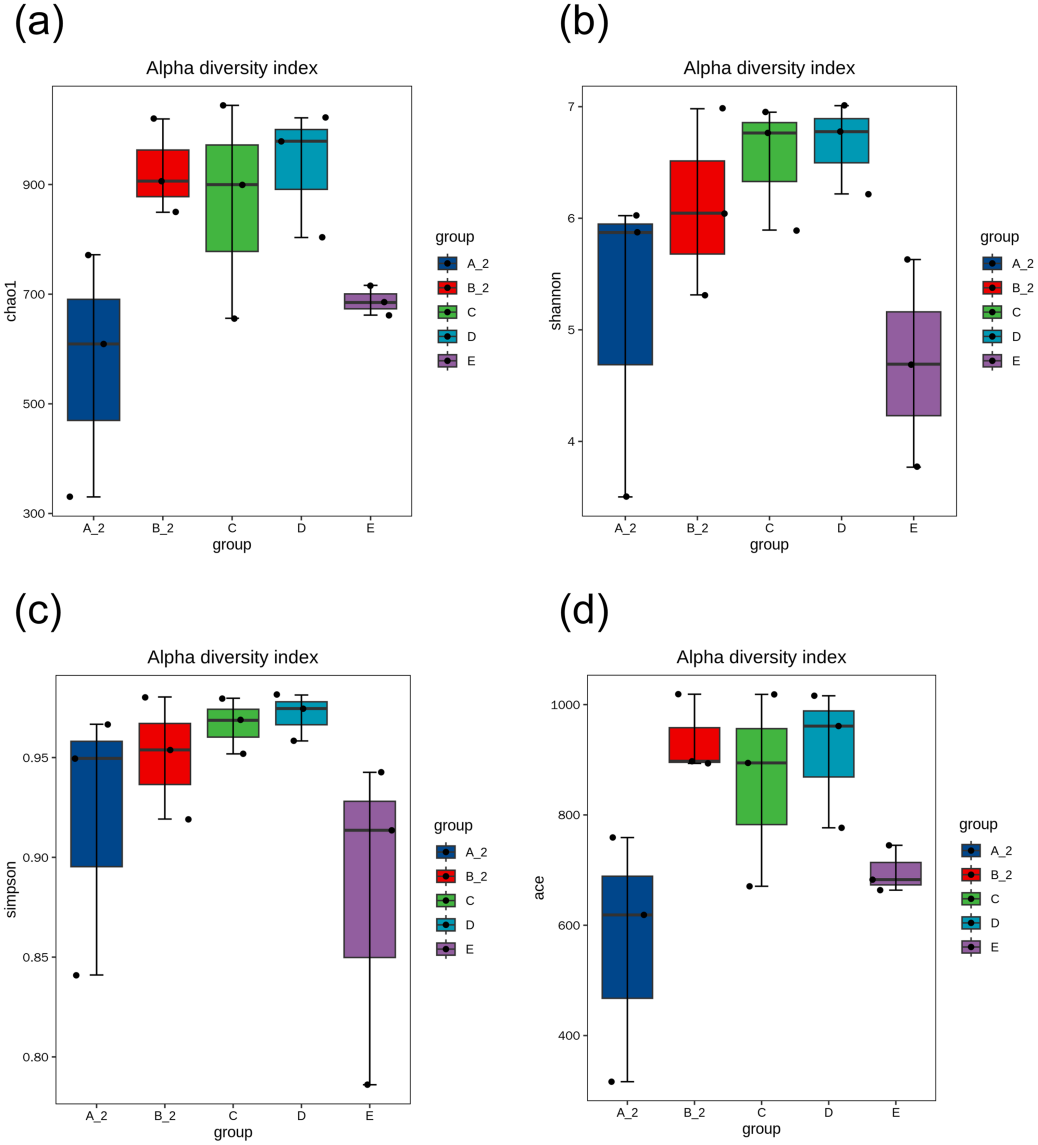
Alpha diversity analysis: **(a)** Chao1 index; **(b)** Shannon index; **(c)** Simpson index; **(d)** ACE index. Group A_2: control-after treatment; Group B_2: model-after treatment; Group C: low-dose JuA; Group D: high-dose JuA; Group E: tiapride. JuA, Jujuboside A.

Alpha diversity analysis revealed that Chao1, Shannon, Simpson, and ACE indices were lower in Group B than in Group A, although these differences were not statistically significant. Compared with those in Group B, Chao1 and ACE indices increased in Groups C and D, whereas Shannon and Simpson indices decreased. In Group E, Chao1 indices decreased, while Shannon and Simpson indices increased compared with those in Group B; however, these differences were not statistically significant. ACE indices in Group E were significantly lower than those in Group B_2 (*p* < 0.05) (Figure 5).

**Figure 6:**
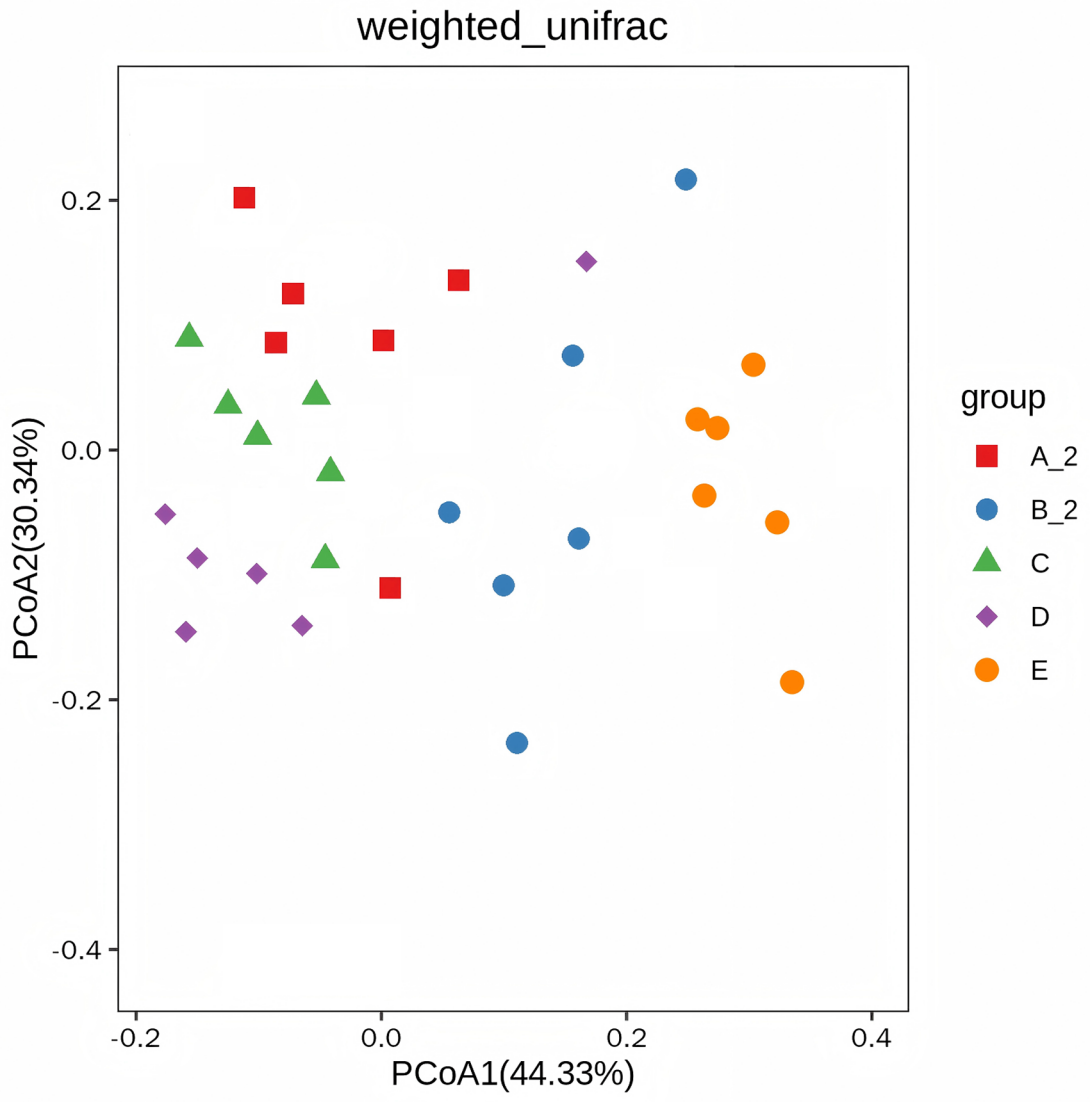
Beta diversity analysis. Group A_2: control-after treatment; Group B_2: model-after treatment; Group C: low-dose JuA; Group D: high-dose JuA; Group E: tiapride. JuA, Jujuboside A.

Beta diversity was evaluated using Principal Coordinate Analysis (PCoA) based on weighted UniFrac distances. The PCoA plot indicated distinct structural shifts in the gut microbiome following TD induction (Figure 6).

**Figure 7:**
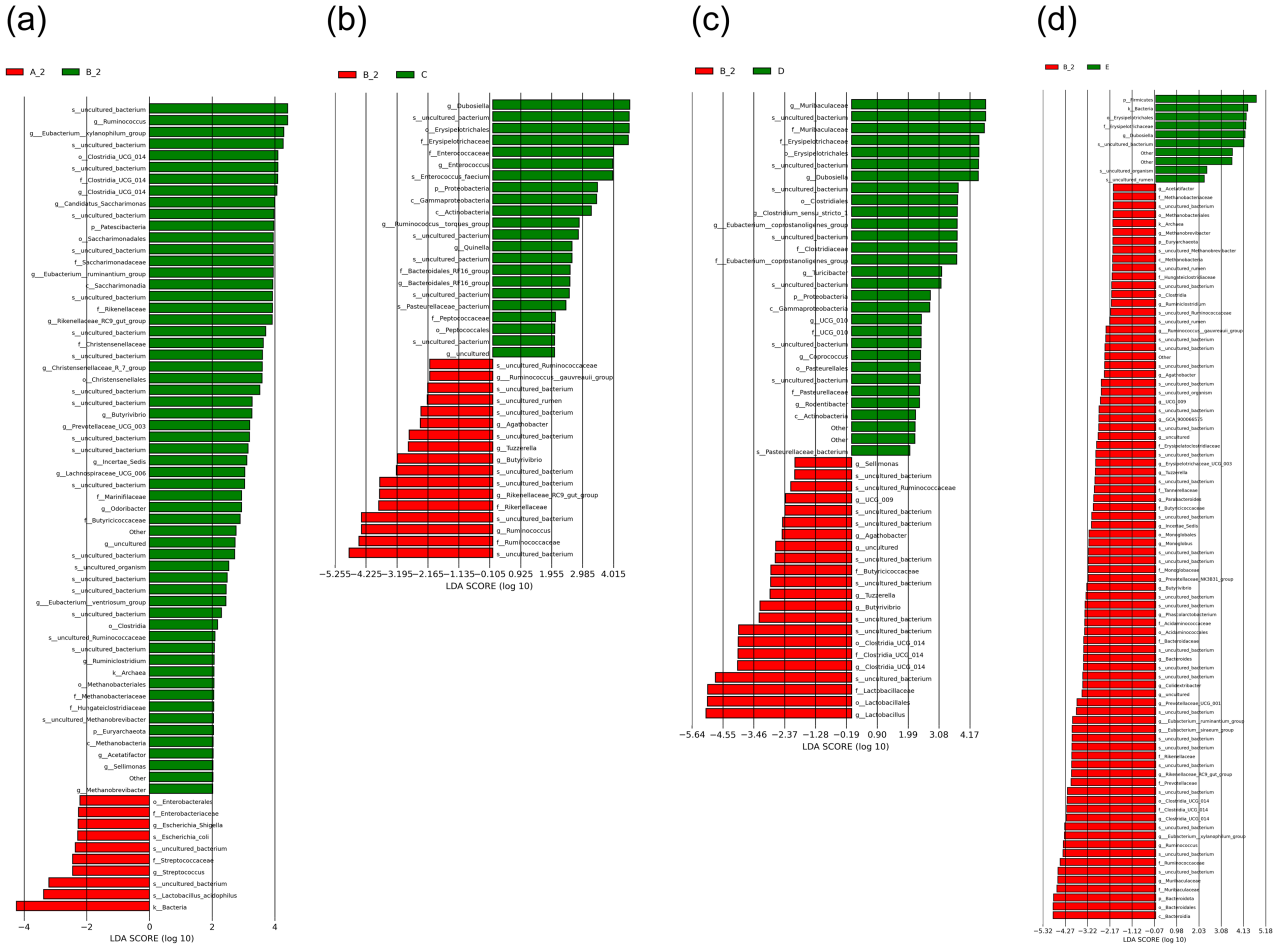
LDA scores between groups. LDA diagram of LEfSe analysis, the absolute value of LDA was set at 3.0: **(a)** Group A_2 vs. B_2; **(b)** Group B_2 vs. C; **(c)** Group B_2 vs. D; **(d)** Group B_2 vs. E. Group A_2: control-after treatment; Group B_2: model-after treatment; Group C: low-dose JuA; Group D: high-dose JuA; Group E: tiapride. JuA, Jujuboside A; LDA, linear discriminant analysis; LEfSe, linear discriminant analysis effect size.

Linear discriminant analysis scores was used to identify differentially abundant taxa. Figure 7 illustrates significant phylogenetic differences in the gut microbiome among groups.

### Differentially expressed genes related to the intestinal microbiota

**Figure 8:**
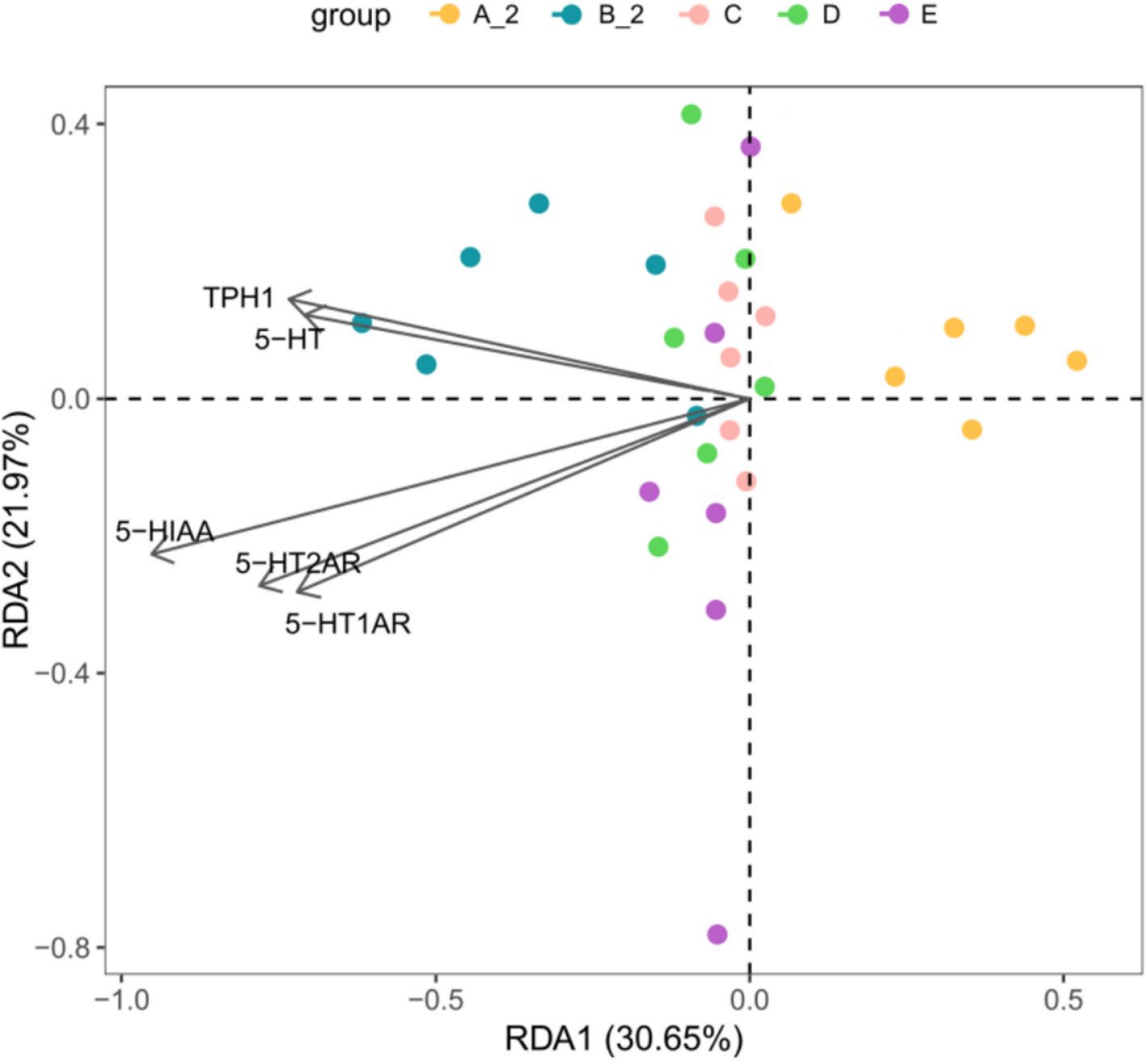
Differentially expressed genes related to the gut microbiota in groups. RDA analysis. Group A_2: control-after treatment; Group B_2: model-after treatment; Group C: low-dose JuA; Group D: high-dose JuA; Group E: tiapride. JuA, Jujuboside A; RDA, redundancy analysis.

Redundancy analysis (RDA) was performed to explore relationships between genus-level microbiota composition and differentially expressed genes. Factors including *TPH1*, *5-HT*, *5-HIAA*, *5-HT1AR*, and *5-HT2AR* were used as environmental variables in the RDA. The environmental factors (*TPH1*, *5-HT*, *5-HIAA*, *5-HT1AR* and *5-HT2AR*) clustered on the same side as intestinal microbiota of TD model groups, indicating that these factors correlated with the microbial composition of rats with TD. The RDA plot demonstrated clear separation between Groups B and A along the first ordination axis, which was strongly associated with 5-HT signaling vectors. The clustering of TD samples opposite to the direction of these vectors visually confirms the downregulation of the intestinal 5-HT system in TD rats. Notably, microbial communities in the JuA-treated groups (C and D) shifted away from the model cluster toward the normal group. This suggests that JuA partially restores gut microbiome structure, a recovery correlated with restored 5-HT signaling (Figure 8).

## Discussion

The pathogenesis of TD remains unclear, although studies implicate the contribution of genetic, immunological, neurochemical, and psychological factors (Deeb et al., 2019; Kim et al., 2019). The gastrointestinal tract is densely innervated by the CNS, autonomic nervous system, and enteric nervous system (Doifode et al., 2021; Cryan et al., 2019). Gastrointestinal sensory information is transmitted to the brain via the ENS or vagal afferents; concurrently, gut microorganisms modulate the development and function of enteric nerves by activating Toll-like receptors (Cryan et al., 2019; Muller et al., 2020). The gut microbiota influences 5-HT release, thereby altering host behavior and cognition. 5-HT is a neurotransmitter found in the CNS and digestive tract (Ma et al., 2023). The gut microbiota regulates its synthesis, metabolism, and physiological functions (Yano et al., 2015). 5-HT levels are significantly reduced in the blood and colon of germ-free rats. Upon microbial colonization, 5-HT levels normalize (Strandwitz, 2018). Moreover, the gut microbiota may mediate gastrointestinal symptoms in children with autism spectrum disorder via the 5-HT pathway (Zhang et al., 2021).

In this study, we examined the expression of 5-HT and its metabolite, (5-HIAA) in rats with TD. JuA alleviated colonic pathology in rats with TD, increasing goblet cell numbers and ameliorating glandular damage. JuA treatment upregulated 5-HT and 5-HIAA levels. Furthermore, intestinal expression of TPH, the rate-limiting enzyme for 5-HT synthesis, increased. These findings suggest that JuA elevates colonic 5-HT levels, potentially exerting its therapeutic effect by upregulating TPH1 expression.

The gut microbiota of rats with TD differs from that of healthy controls (Xi et al., 2021; Wang et al., 2022). Fecal microbiota transplantation has been reported to improve the intestinal environment, alleviate clinical symptoms, and increase serum 5-HT levels in rats with TD, suggesting that fecal microbiota transplantation mitigates TD progression by promoting 5-HT secretion (Li et al., 2022). To investigate the impact of JuA on the gut microbiota, 16S rRNA gene sequencing was performed on fecal samples from treated rats.

The present study found that JuA and tiapride exerted distinct, often opposing, effects on gut microbiota composition. In rats with TD, the relative abundance of of *Bacilli*, *Lactobacillaceae*, *Lactobacillus*, *Lactobacillales* and *Firmicutes* were decreased. JuA treatment increased the abundance of *Bacteroidia* (including *Muribaculaceae*, *Bacteroidales* and *Bacteroidota*) while reducing *Bacilli*, *Lactobacillus* lineage and *Firmicutes*. Conversely, tiapride increased the abundance of *Bacilli*, *Lactobacillaceae*, *Lactobacillus*, *Lactobacillales* and *Firmicutes*, and reduced the *Bacteroidota* lineage.

*Lactobacillaceae* are beneficial bacteria; alterations in their abundance can impact CNS immune function (Montgomery et al., 2024). *Muribaculaceae* produce short-chain fatty acids by fermenting endogenous and exogenous polysaccharides (Zhu et al., 2024). JuA treatment enriched *Muribaculaceae*, whereas tiapride enriched *Lactobacillaceae* and *Firmicutes*. Although *Lactobacillus* is generally considered beneficial, its reduced abundance following JuA treatment does not necessarily indicate a detrimental effect. Microbial function is highly context-dependent and involves complex inter-species interactions within the gut ecosystem. The therapeutic effects of JuA may arise from the overall restructuring of the microbiota, particularly the enrichment of *Muribaculaceae* and other short-chain fatty acid-producing taxa, which have been associated with enhanced intestinal barrier function and modulation of neurotransmitter synthesis (Zhu et al., 2024; Fusco et al., 2023). While both JuA and tiapride attenuated stereotyped behaviors in TD rats, their effects on the gut microbiota were distinct suggesting that different microbial configurations may lead to similar therapeutic outcomes (Jiang et al., 2025). Alpha diversity, beta diversity, and taxonomic profiles differed between TD model rats and healthy controls. JuA and tiapride administration induced structural shifts in the gut microbiota of TD rats; however, the direction of these shifts diverged. Importantly, the RDA results integrate our key findings by demonstrating a strong correlation between the specific gut microbial community structure of the TD model groups and the downregulation of the intestinal 5-HT signaling system.

An important question arising from our findings is whether JuA exerts its modulatory effect on the gut microbiota directly, as a prebiotic compound, or indirectly through host absorption and subsequent systemic effects. JuA, like many saponins, may have low oral bioavailability (Li et al., 2024), suggesting that a substantial portion of the administered dose reaches the large intestine, where it could directly interact with the microbial community. This direct interaction might selectively enrich specific bacterial taxa, such as *Muribaculaceae*, while inhibiting others. Conversely, the absorbed fraction of JuA is known to cross the blood–brain barrier and modulate neurotransmission (Zhang et al., 2018), which could alter gut physiology via the brain-gut axis, thereby indirectly shaping the microbial environment (Loh et al., 2024). It is plausible that both mechanisms operate in concert. JuA may initiate changes through direct microbicidal or prebiotic activity, and these microbial shifts could be perpetuated and amplified by host physiological changes mediated by the absorbed compound. Future studies employing fecal microbiota transplantation from JuA-treated donors into germ-free or antibiotic-treated TD rats could help delineate the causal contribution of the direct vs. indirect pathways.

This study has some limitations. First, the sample size was relatively small (*n* = 6 per group), which may limit statistical power and generalizability of the findings. Although significant differences were observed in key outcomes, the small cohort size increases the risk of type II errors and may reduce the reproducibility of the results. Therefore, future studies with larger sample sizes are warranted to validate our findings. Second, as the study focused exclusively on male rats, the findings may not be fully generalizable to females, given known sex differences in TD prevalence and manifestation. Third, this was a correlational study. Although RDA indicated an association between microbial composition and 5-HT markers, it did not establish causality; further experiments are required to confirm mechanistic links. Fourth, due to tissue sample limitations, we were unable to measure 5-HT levels in plasma or brain regions such as the striatum, assess intestinal barrier function markers including zonula occludens-1, occludin, and claudin, validate TPH1 protein expression by Western blot, or quantify short-chain fatty acids, future studies incorporating these analyses will be important to more comprehensively elucidate the gut-brain axis involvement in JuA’s therapeutic effects. Fifth, although this study identified distinct taxonomic shifts associated with JuA treatment, we did not perform functional prediction or permutational multivariate analysis of variance statistical validation for beta diversity, future studies should incorporate these approaches. Additionally, this study assessed only the short-term effects of JuA; long-term impacts and potential side effects warrant further exploration. Finally, the translational relevance of these findings to human TD patients requires validation through clinical trials.

## Conclusion

JuA attenuated abnormal activity and stereotyped behaviors in TD rats. Furthermore, JuA upregulated 5-HT, 5-HIAA, and TPH1 levels and normalized the gut microbiota. We conclude that JuA may exert its therapeutic effects at least in part through remodeling gut microbiota and enhancing intestinal 5-HT signaling. This study highlights JuA as a potential therapeutic candidate for TD management.

## Data Availability

All data generated or analyzed during this study are included in the published article and its figures/tables. No additional data have been deposited in an external repository. Further inquiries can be directed to the corresponding author.

## References

[ref1] Al Kadasah S. Al Mutairy A. Siddiquei M. Khan H. A. Abdulwahid Arif I. Al Moutaery K. . (2009). Pentoxifylline attenuates iminodipropionitrile-induced behavioral abnormalities in rats. Behav. Pharmacol. 20, 356–360. doi: 10.1097/FBP.0b013e32832ec5ea, 19584713

[ref2] Cai Z. Che C. Li D. Li X. Yu X. Yu L. . (2025). Common gut microbial signatures in autism Spectrum disorder and attention deficit hyperactivity disorder. Autism Res. 18, 741–751. doi: 10.1002/aur.70016, 40045873

[ref3] Cryan J. F. O'Riordan K. J. Cowan C. S. M. Sandhu K. V. Bastiaanssen T. F. S. Boehme M. . (2019). The microbiota-gut-brain axis. Physiol. Rev. 99, 1877–2013. doi: 10.1152/physrev.00018.2018 31460832

[ref4] Cui W. Guo Z. Chen X. Yan R. Ma W. Yang X. . (2024). Targeting modulation of intestinal flora through oral route by an antimicrobial nucleic acid-loaded exosome-like nanovesicles to improve Parkinson's disease. Sci. Bull. (Beijing) 69, 3925–3935. doi: 10.1016/j.scib.2024.10.027, 39500690

[ref5] Deeb W. Malaty I. A. Mathews C. A. (2019). Tourette disorder and other tic disorders. Handb. Clin. Neurol. 165, 123–153. doi: 10.1016/b978-0-444-64012-3.00008-3, 31727209

[ref6] Diamond B. I. Reyes M. G. Borison R. (1982). A new animal model for Tourette syndrome. Adv. Neurol. 35, 221–225. 6959491

[ref7] Doifode T. Giridharan V. V. Generoso J. S. Bhatti G. Collodel A. Schulz P. E. . (2021). The impact of the microbiota-gut-brain axis on Alzheimer's disease pathophysiology. Pharmacol. Res. 164:105314. doi: 10.1016/j.phrs.2020.105314 33246175

[ref8] Fan F. Han F. Hao L. (2022). Mechanisms of action of semen Ziziphi Spinosae in the treatment of Tourette syndrome. Degener. Neurol. Neuromuscul. Dis. 12, 85–96. doi: 10.2147/DNND.S370278, 35875687 PMC9297330

[ref9] Fan F. Zhang S. Wu H. Zhang M. Han F. (2025). Modulatory effects of Jujuboside a on amino acid neurotransmitter profiles in tic disorder. Brain Behav. 15:e71041. doi: 10.1002/brb3.71041, 41220182 PMC12606026

[ref10] Fusco W. Lorenzo M. B. Cintoni M. Porcari S. Rinninella E. Kaitsas F. . (2023). Short-chain fatty-acid-producing bacteria: key components of the human gut microbiota. Nutrients 15:2211. doi: 10.3390/nu15092211, 37432351 PMC10180739

[ref11] Hao Z. Lu Y. Hao Y. Luo Y. Wu K. Zhu C. . (2024). Fungal mycobiome dysbiosis in choledocholithiasis concurrent with cholangitis. J. Gastroenterol. 60, 340–355. doi: 10.1007/s00535-024-02183-y, 39604579

[ref12] He J. Jin Y. He C. Li Z. Yu W. Zhou J. . (2023). Danggui Shaoyao San: comprehensive modulation of the microbiota-gut-brain axis for attenuating Alzheimer's disease-related pathology. Front. Pharmacol. 14:1338804. doi: 10.3389/fphar.2023.1338804, 38283834 PMC10811133

[ref13] Jiang K. Pang X. Li W. Xu X. Yang Y. Shang C. . (2025). Interbacterial warfare in the human gut: insights from Bacteroidales' perspective. Gut Microbes 17:2473522. doi: 10.1080/19490976.2025.2473522, 40038576 PMC11901371

[ref14] Kim S. Greene D. J. Bihun E. C. Koller J. M. Hampton J. M. Acevedo H. . (2019). Provisional tic disorder is not so transient. Sci. Rep. 9:3951. doi: 10.1038/s41598-019-40133-4, 30850688 PMC6408476

[ref15] Knight T. Steeves T. Day L. Lowerison M. Jette N. Pringsheim T. (2012). Prevalence of tic disorders: a systematic review and meta-analysis. Pediatr. Neurol. 47, 77–90. doi: 10.1016/j.pediatrneurol.2012.05.002 22759682

[ref16] Li H. Wang Y. Zhao C. Liu J. Zhang L. Li A. (2022). Fecal transplantation can alleviate tic severity in a Tourette syndrome mouse model by modulating intestinal flora and promoting serotonin secretion. Chin. Med. J. 135, 707–713. doi: 10.1097/cm9.0000000000001885, 35288507 PMC9276343

[ref17] Li W. Zhang Y. Zhao J. Yang T. Xie J. (2024). L-carnitine modified nanoparticles target the OCTN2 transporter to improve the oral absorption of jujuboside B. Eur. J. Pharm. Biopharm. 196:114185. doi: 10.1016/j.ejpb.2024.114185 38280469

[ref18] Loh J. S. Mak W. Q. Tan L. K. S. Ng C. X. Chan H. H. Yeow S. H. . (2024). Microbiota-gut-brain axis and its therapeutic applications in neurodegenerative diseases. Signal Transduct. Target. Ther. 9:37. doi: 10.1038/s41392-024-01743-1, 38360862 PMC10869798

[ref19] Ma J. Wang R. Chen Y. Wang Z. Dong Y. (2023). 5-HT attenuates chronic stress-induced cognitive impairment in mice through intestinal flora disruption. J. Neuroinflammation 20:23. doi: 10.1186/s12974-023-02693-1, 36737776 PMC9896737

[ref20] Martino D. Pringsheim T. M. (2018). Tourette syndrome and other chronic tic disorders: an update on clinical management. Expert. Rev. Neurother. 18, 125–137. doi: 10.1080/14737175.2018.1413938, 29219631

[ref21] Montgomery T. L. Toppen L. C. Eckstrom K. Heney E. R. Kennedy J. J. Scarborough M. J. . (2024). *Lactobacillaceae* differentially impact butyrate-producing gut microbiota to drive CNS autoimmunity. Gut Microbes 16:2418415. doi: 10.1080/19490976.2024.2418415, 39462277 PMC11520542

[ref22] Muller P. A. Schneeberger M. Matheis F. Wang P. Kerner Z. Ilanges A. . (2020). Microbiota modulate sympathetic neurons via a gut-brain circuit. Nature 583, 441–446. doi: 10.1038/s41586-020-2474-7, 32641826 PMC7367767

[ref23] Müller-Vahl K. R. Szejko N. Verdellen C. Roessner V. Hoekstra P. J. Hartmann A. . (2022). European clinical guidelines for Tourette syndrome and other tic disorders: summary statement. Eur. Child Adolesc. Psychiatry 31, 377–382. doi: 10.1007/s00787-021-01832-4, 34244849 PMC8940881

[ref24] Olsen I. Hicks S. D. (2020). Oral microbiota and autism spectrum disorder (ASD). J. Oral Microbiol. 12:1702806. doi: 10.1080/20002297.2019.1702806, 31893019 PMC6913665

[ref25] Park H. J. Lee S. E. Kim H. B. Isaacson R. E. Seo K. W. Song K. H. (2015). Association of obesity with serum leptin, adiponectin, and serotonin and gut microflora in beagle dogs. J. Vet. Intern. Med. 29, 43–50. doi: 10.1111/jvim.12455, 25407880 PMC4858068

[ref26] Pringsheim T. Holler-Managan Y. Okun M. S. Jankovic J. Piacentini J. Cavanna A. E. . (2019a). Comprehensive systematic review summary: treatment of tics in people with Tourette syndrome and chronic tic disorders. Neurology 92, 907–915. doi: 10.1212/wnl.0000000000007467, 31061209 PMC6537130

[ref27] Pringsheim T. Okun M. S. Müller-Vahl K. Martino D. Jankovic J. Cavanna A. E. . (2019b). Practice guideline recommendations summary: treatment of tics in people with Tourette syndrome and chronic tic disorders. Neurology 92, 896–906. doi: 10.1212/WNL.0000000000007466, 31061208 PMC6537133

[ref28] Sanger G. J. (2008). 5-hydroxytryptamine and the gastrointestinal tract: where next? Trends Pharmacol. Sci. 29, 465–471. doi: 10.1016/j.tips.2008.06.008, 19086255

[ref29] Stiede J. T. Woods D. W. (2020). Pediatric prevention: tic disorders. Pediatr. Clin. N. Am. 67, 547–557. doi: 10.1016/j.pcl.2020.02.009, 32443993

[ref30] Strandwitz P. (2018). Neurotransmitter modulation by the gut microbiota. Brain Res. 1693, 128–133. doi: 10.1016/j.brainres.2018.03.015, 29903615 PMC6005194

[ref31] Tagi V. M. Tosi M. Greco I. P. Stucchi E. Verduci E. Zuccotti G. (2024). Pediatric autoimmune neuropsychiatric disorders associated with streptococcal infections and gut microbiota composition: what do we know? Front. Nutr. 11:1477893. doi: 10.3389/fnut.2024.1477893, 39834450 PMC11743372

[ref32] Tagwerker Gloor F. Walitza S. (2016). Tic disorders and Tourette syndrome: current concepts of etiology and treatment in children and adolescents. Neuropediatrics 47, 084–096. doi: 10.1055/s-0035-1570492, 26829367

[ref33] Wang Y. Xu H. Jing M. Hu X. Wang J. Hua Y. (2022). Gut microbiome composition abnormalities determined using high-throughput sequencing in children with tic disorder. Front. Pediatr. 10:831944. doi: 10.3389/fped.2022.831944, 35601424 PMC9114666

[ref34] Wang Y. Zhu J. Zou N. Zhang L. Wang Y. Zhang M. . (2023). Pathogenesis from the microbial-gut-brain axis in white matter injury in preterm infants: a review. Front. Integr. Neurosci. 17:1051689. doi: 10.3389/fnint.2023.1051689, 37006416 PMC10060642

[ref35] Xi W. Gao X. Zhao H. Luo X. Li J. Tan X. . (2021). Depicting the composition of gut microbiota in children with tic disorders: an exploratory study. J. Child Psychol. Psychiatry 62, 1246–1254. doi: 10.1111/jcpp.13409, 33738808

[ref36] Yael D. Vinner E. Bar-Gad I. (2015). Pathophysiology of tic disorders. Mov. Disord. 30, 1171–1178. doi: 10.1002/mds.26304, 26179434

[ref37] Yang C. Cheng X. Zhang Q. Yu D. Li J. Zhang L. (2020). Interventions for tic disorders: an updated overview of systematic reviews and meta analyses. Psychiatry Res. 287:112905. doi: 10.1016/j.psychres.2020.112905, 32163785

[ref38] Yang R. Li Z. Dong L. Heng Y. Song L. Guo L. . (2025). Analysis of components in Ziziphi Spinosae semen before and after processing based on targeted and untargeted metabolomics. Foods 14:3771. doi: 10.3390/foods14213771, 41227741 PMC12607712

[ref39] Yano J. M. Yu K. Donaldson G. P. Shastri G. G. Ann P. Ma L. . (2015). Indigenous bacteria from the gut microbiota regulate host serotonin biosynthesis. Cell 161, 264–276. doi: 10.1016/j.cell.2015.02.047, 25860609 PMC4393509

[ref40] Zhang M. Qian C. Zheng Z.-G. Qian F. Wang Y. Thu P. M. . (2018). Jujuboside a promotes aβ clearance and ameliorates cognitive deficiency in Alzheimer's disease through activating Axl/HSP90/PPARγ pathway. Theranostics 8, 4262–4278. doi: 10.7150/thno.26164, 30128052 PMC6096387

[ref41] Zhang Z. Zhang Y. Li J. Fu C. Zhang X. (2021). The neuroprotective effect of tea polyphenols on the regulation of intestinal Flora. Molecules 26:34204244. doi: 10.3390/molecules26123692, 34204244 PMC8233780

[ref42] Zhu Y. Chen B. Zhang X. Akbar M. T. Wu T. Zhang Y. . (2024). Exploration of the *Muribaculaceae* family in the gut microbiota: diversity, metabolism, and function. Nutrients 16:39203797. doi: 10.3390/nu16162660, 39203797 PMC11356848

[ref43] Zhu L. Ma S. Li K. Xiong P. Qin S. Cai W. (2022). Systematic screening of chemical constituents in the traditional Chinese medicine Arnebiae Radix by UHPLC-Q-Exactive Orbitrap mass spectrometry. Molecules 27:2631. doi: 10.3390/molecules27092631, 35565981 PMC9104353

